# Medical emergency motorcycle – is it useful in a Scandinavian Emergency Medical Service?

**DOI:** 10.1186/1757-7241-17-9

**Published:** 2009-02-24

**Authors:** Anders Rostrup Nakstad, Bjørn Bjelland, Mårten Sandberg

**Affiliations:** 1 The Air Ambulance department, Oslo University Hospital – Ullevål, Oslo, Norway; 2 Department of Anaesthesia, Oslo University Hospital – Ullevål, Oslo, Norway; 3 Oslo and Akershus Ambulance Service, Oslo University Hospital – Ullevål, Oslo, Norway

## Abstract

**Background:**

Medical emergency motorcycles (MEM) can be used in time-critical conditions like cardiac arrest and multi-traumatized patients in an attempt to reduce the response time. Other potential benefits with MEM are more efficient patient evaluation, reduction of unnecessary EMS car ambulance missions and reduced cost. The potential benefits have been evaluated in this study. The incidence of accidents when operating the vehicle was also of interest.

**Methods:**

A prospective study was performed when MEM was introduced as a trial in an urban ambulance service in Norway.

**Results:**

A total of 703 MEM missions were registered in the period. The mean emergency driving time was significantly shorter for the MEM than for the ambulance car located at the same station (6 min 24 seconds vs. 6 min 54 seconds). In addition to time-critical conditions, the MEM was used to evaluate patients when the need for emergency medical assistance was uncertain, and this practice lead to a reduced number of unnecessary car ambulance missions. No accidents involving the MEM were registered in the study period. The hourly cost of running the MEM was € 29 vs. € 75 for a car ambulance. However, the actual cost benefit is smaller since the weather conditions make it impossible to run a MEM in wintertime.

**Conclusion:**

The small reduction in driving time when using a MEM instead of a car ambulance was statistically significant but probably of little clinical importance. The number of unnecessary car ambulance missions was reduced. It was cheaper to operate a MEM than a car ambulance, but the cost-effectiveness was reduced since the MEM could not operate 12 months a year. The lack of accidents may be contributed to the extensive training of the drivers and the fact that the vehicle was operated in daylight only.

## Introduction

In time-critical disorders like cardiac arrest, myocardial infarction, severe respiratory disease and polytrauma immediate response from the Emergency Medical Service (EMS) is crucial and the fastest mean of transport to the patient must be chosen [1,2]. Recently, it has been focused on the relatively long response times for car ambulances in urban traffic, and the use of medical emergency motorcycles (MEM) has been advocated. In a study from Taiwan, Lin and co-workers demonstrated that a motorcycle had a significantly shorter response time than a regular ambulance [3]. Soares-Oliveira and co-workers recently described the use of MEM in Portugal with emphasis on its efficiency in reducing response times and in evaluating patients where the need for immediate assistance was uncertain [4]. One serious injury and two minor injuries to the MEM paramedics were described in another Portuguese study including 3626 missions [5]. However, the literature about MEM is scarce and motorcycle ambulances are not extensively employed [6].

In the Oslo and Akershus Ambulance Service in Norway, a paramedic manned MEM was introduced as a trial in order to investigate whether the MEM was a time- and cost-efficient supplement to the car ambulances in the service. One aim of the study presented here was to evaluate how the vehicle was used and if it reached patients with potential critical illness faster than the car ambulance did. We also wanted to clarify if the number of unnecessary car ambulance missions was reduced. Furthermore, because of the inherent risk of motorcycle riding, the safety of the new vehicle was studied. Finally, the costs of running a MEM was calculated and compared to the cost of a car ambulance.

## Methods

The study included all MEM missions from May to the end of September 2007. The regional ethical board approved the study. Paramedics with long clinical experience manned the MEM, and the vehicle was equipped with a defibrillator, standard drugs, oxygen, suction device and airway management equipment. Data including driving time, dispatch reason, patient characteristics and treatment were collected. The vehicle operated 15 hours a day, six days a week in the city and it was co-localized with a car ambulance. Data from this car ambulance was used to compare the driving time of the two different types of vehicles, although they were not routinely used in the same missions. The operators at the dispatch centre registered their criteria for use of the MEM in the first two months of the period while the MEM paramedics provided operational and medical information about the missions in the whole five-month period. Data were collected using the spreadsheet Excel (Microsoft, Redmond, WA), and statistical analysis was performed with EPI-info (Centre for Disease Control (CDC), WHO) by use of the non-parametric Mann-Whitney/Wilcoxon two-sample test.

## Results

The vehicle was used both in time-critical missions and to clarify the need for further emergency medical service (table 1). A total of 703 MEM missions were registered in the study period, including 60 non-patient missions where the MEM was used to cover areas in the city with a temporary shortage of ambulance. Thus the MEM initiated 643 missions to a potential patient site. A total of 585 (91.0%) of these missions were completed, while the remaining 58 (9.0%) were aborted because of updated information that emergency assistance was not needed. In 292 (49.9%) of the 585 completed missions, a total of 298 persons with a potential medical problem were examined. The mean age of the patients was 51.6 years and 56% were male. Various medical disorders in stable patients, trauma and neurological disease accounted for more than half of the problems (table 2). In the remaining 293 (50.1%) of missions there was no evident patient injury or illness.

**Table 1:** Dispatch criteria for use of MEM

	**n**	**%**
Closest vehicle to patient site	108	33.8

Sent to clarify need for transportation	107	33.4

Sent to assist car ambulance	55	17.2

Motorcycle only available unit in the area	17	5.3

Most suitable vehicle for reaching patient site	10	3.1

Other reason	23	7.2

	320	100.0

The criteria used by the operators when dispatching the MEM in 320 missions during a two month period.

**Table 2:** Main medical problem in MEM missions

	**n**	**%**
Medical problem in stable patient	41	13.8

Intoxication with heroine	31	10.4

Suspected stroke	29	9.7

Fractured proximal end of femur	27	9.1

Trauma due to traffic accident, fall or violence	25	8.4

Suspected myocardial infarction	21	7.0

Convulsions (fever in children excluded)	20	6.7

Cardiac arrest	20	6.7

Abdominal pain	17	5.7

Suspected pulmonary disease	14	4.7

Intoxication (heroine excluded)	12	4.0

Psychiatric disease	11	3.7

Minor wound or burn injury	7	2.3

Suspected heart failure	7	2.3

Child with fever	6	2.0

Symptoms related to pregnancy	4	1.3

Other symptom	4	1.3

Convulsions (suspected due to fever in child)	2	0.7

	298	100.0

Main medical problem or symptom presented by the patient – as identified by the paramedic on site.

The operators at the dispatch centre rated 436 (67.8%) of the 643 missions as emergency missions and the average driving time for the MEM in these missions was 6 minutes 24 seconds (SD 4 minutes 14 seconds). For the car ambulance located at the same station the mean response time in the same period during 583 emergency missions was 6 minutes 54 seconds (SD 4 minutes 58 seconds). The 30 seconds time difference between MEM and car ambulance was statistically significant (p = 0.046). In the 282 cases when both the MEM and the ambulance were dispatched to the same patient site, the MEM was first on site in 244 (85%) of the missions.

In 31 of the emergency missions the MEM paramedic cancelled a simultaneously alerted car ambulance since evaluation of the patient indicated that the patient had no need for ambulance transport. In 107 of the missions with the objective to investigate the actual need of emergency care, no indication for ambulance transport was found. In the majority of these cases either a physician was dispatched to the patient to perform a clinical assessment or the patient was transported by taxi to the health care centre. In total, 138 car ambulance missions were avoided because of the use of the MEM. This constitutes 23.5% of the 585 MEM missions to a potential patient site.

The MEM paramedic performed 243 medical interventions in 121 patients before the arrival of another ambulance. Intravenous drugs were given in 63 cases, including 23 cases of naloxone administration to heroine intoxicated patients. Airway management procedures (oropharyngeal tube and/or bag-mask ventilation) were performed in 13 cases. Three patients with cardiac arrest were resuscitated by the MEM paramedic prior to arrival of other health resources. Return of spontaneous circulation (ROSC) was achieved in two of them. In another 17 cardiac arrest cases, the MEM paramedic assisted the car ambulance paramedics that had initiated the resuscitation of the patients.

The cost of starting up the MEM service was calculated to € 90,000. In addition the technical cost of running the vehicle during the five month period was € 50,000. Thus the total cost in the first year of service was € 140,000, while a prolongation of the service would have resulted in an estimated annual cost of € 60,000. The cost for running a car ambulance with two paramedics 24H all week is approximately € 655,000. When the operating hours and the number of months the vehicles were available each year was included, the hourly cost was estimated to € 29 for the MEM and € 75 for the car ambulance.

No accidents involving the MEM were reported in the study period.

## Discussion

This study has some methodological limitations. When comparing the response time of the MEM with a car ambulance one would ideally dispatch both units simultaneously from the same position to the patient site. This was not possible during the study period. As a substitute, we compared the MEM driving times with the driving times in missions performed by the car ambulance operating from the same station in the same period. The 30 seconds difference in mean driving time was statistically significant, although it is highly likely that such a small difference will have little if any effect on patient outcome. The surprisingly small difference may be because of less rush traffic in Oslo than in larger cities. It may also result from the fact that the MEM and the car ambulance did not start from the same location in most missions.

The reduction in number of car ambulance missions due to the evaluation performed by the MEM was substantial when keeping the total number of MEM missions in mind. This way the MEM can increase the availability of a paramedic even though the MEM itself cannot transport patients. On the other hand, using qualified paramedics on a motorcycle decreases the possibility to maximize the number of car ambulances and thereby decreases the total transport capacity of the service.

In approximately half of the missions the MEM paramedic did not attended any patients. Based on these numbers there seems to be a potential for improving the quality of the initial medical triage performed by the dispatch centre operator. It must be kept in mind, however, that the MEM was intentionally used to evaluate cases where the need of ambulance transport was unclear. Thus the number of missions without patients will be higher for the MEM than for other vehicles.

Collaboration between at least two health care professionals is important in conditions like cardiac arrest. That fact is not, however, an argument against MEM since in such instances a MEM paramedic can assist the paramedics from the car ambulance, or vice versa. In this study the MEM assisted car ambulances in 17 instances of cardiac arrest and the MEM paramedic also assisted the car ambulance paramedics in other cases like carrying heavy patients down staircases. Thus the ability to quickly assist other ambulances seems to be a good argument for using MEM.

Since the difference in response time between the two vehicles was clinically insignificant, it is the cost that eventually will decide whether it is sensible to implement a MEM in an EMS system. The cost pr. hour for a MEM was € 29 compared to € 75 for a car ambulance. In countries like Norway with a harsh climate, weather conditions will make a MEM unsafe for a substantial part of the year. Thus, a MEM can only supplement a car ambulance and not replace it and it is therefore not a cost-effective solution.

The MEM was not used after 10 PM in order to reduce the accident risk. No injuries to the MEM paramedic were registered and this is in accordance with Portuguese results indicating 0.8 injuries pr. 1,000 MEM missions, supporting the idea that a MEM service can be run with a good safety record provided that the paramedics have sufficient training and safety equipment.

## Conclusion

In an urban service like ours, a MEM may lead to a statistically significant reduction in response times, but the clinical impact is small. A MEM may be useful as a supplement to the car ambulance service in conditions like cardiac arrest where it is beneficial to have more than two paramedics at the site. Furthermore, the MEM paramedic can evaluate the need for further emergency treatment in unclear situations. The cost pr. hour for a MEM is significantly lower than for a car ambulance, but that benefit is partly lost if the MEM cannot be operated the whole year. No injuries to the MEM paramedic were registered in the study period.

## Competing interests

The authors declare that they have no competing interests.

## Authors' contributions

ARN participated in the design of the study, the sampling of data, the statistical analysis and the writing of the manuscript. BB participated in the design of the study, the sampling of data and the writing of the manuscript. MS participated in the design of the study, the statistical analysis and the writing of the manuscript.
